# A Case of Hypophosphatasia With Normal Alkaline Phosphatase Levels

**DOI:** 10.1016/j.aace.2023.11.006

**Published:** 2023-11-22

**Authors:** Antara Dattagupta, Steven Petak

**Affiliations:** 1 Division of Endocrinology, Diabetes, and Metabolism, Department of Medicine, Houston Methodist Hospital, Houston, Texas; 2 Department of Medicine, Washington University School of Medicine, St. Louis, Missouri

**Keywords:** hypophosphatasia, ALPL, TNSALP, bone-specific ALP

## Abstract

**Background/Objective:**

Hypophosphatasia (HPP) is a rare disease associated with low serum alkaline phosphatase (ALP) activity. Here, we present a case of a patient with normal serum ALP levels diagnosed with HPP.

**Case Report:**

A 36-year-old woman presented with progressive fatigue, weakness, and joint pain. She had been evaluated in the past for genetic disorders due to these symptoms and was found to have a history of several total ALP levels within normal limits but elevated vitamin B6 levels. She also reported having loose teeth and “gray gums” during her childhood. Bone-specific ALP was tested for suspicion of HPP and returned at 4.4 μ/L (reference range, 5.3-19.5 μg/L), which prompted genetic testing. Genetic testing confirmed a positive pathogenetic variant of the *ALPL* gene, the c.542C>T (p.Ser181Leu) variant. She started asfotase alfa treatment to improve her symptoms.

**Discussion:**

HPP was diagnosed based on clinical suspicion supported by laboratory findings, which can cause it to be underdiagnosed or misdiagnosed. Current literature reports that a low total ALP level is the main biochemical marker of HPP and the only level needed to diagnose the disease. However, bone-specific ALP, a common marker used for bone turnover, has not been required to be tested.

**Conclusion:**

This case highlights a patient with normal total ALP, but low bone-specific ALP diagnosed with HPP confirmed by genetic testing. This case warrants future investigation into the diagnostic approach to HPP and the diagnostic utility between ALP and bone-specific ALP.

**Highlights**

• Normal total alkaline phosphatase levels do not exclude hypophosphatasia (HPP) diagnosis
• Potential expansion of biomarker levels is needed to clinically diagnose HPP
• Asfotase alfa treatment in retroactively diagnosed patients with HPP

**Clinical Relevance**

Currently, a low serum alkaline phosphatase (ALP) is the only biomarker needed to clinically diagnose a patient with suspected hypophosphatasia (HPP). However, patients with normal serum ALP levels like the case described, may have their diagnosis of HPP overlooked. The diagnostic approach to HPP with regard to biomarkers warrants further investigation as well as the utility of biomarkers such as serum ALP and bone-specific ALP.

## Introduction

Hypophosphatasia (HPP) is a rare disease caused by sequence variations in the tissue-nonspecific isoenzyme of the alkaline phosphatase (ALP) gene.^1^ More than 300 sequence variants have been identified, and the disease follows either an autosomal recessive or a dominant transmission.^2^ Serum ALP is a common test reported in the comprehensive metabolic panel requested in both inpatient and outpatient clinical practice. Patients with HPP have decreased ALP levels and may have elevated vitamin B6 levels. However, current literature and studies report that a low total ALP level is the main biochemical marker of HPP and the only marker required to diagnose the disease.^1^^,^^3^, ^4^, ^5^

Here, we report a case of a genetically confirmed symptomatic patient with HPP with total ALP levels within normal limits but a low bone-specific ALP level.

## Case Report

A 36-year-old woman presented with progressive fatigue, weakness, and joint pain. Her medical history was significant for attention-deficit hyperactivity disorder, migraines, and Hashimoto’s disease. She reported having a childhood history of loose teeth, several cavities, and “gray gums.” She had a surgical history significant for partial hysterectomy and several operations on her hands for De Quervain’s tenosynovitis, trigger finger, and cyst removal.

For her presenting symptoms, she was previously evaluated for genetic abnormalities at an outside facility in 2021, where vitamin B6 levels of 301.3 nmol/L and 202.6 nmol/L (reference range, 20-125 nmol/L) approximately 5 months apart were reported. Additionally, in 2021, her total ALP levels were 50 U/L in January, 36 U/L in March, and 56 U/L in July, all within the normal limits of 31 to 125 U/L. Her ALP levels in 2017 and 2020 were 34 U/L and 43 U/L, respectively (Table). In 2022, she underwent a bone dual-energy x-ray absorptiometry examination at an outside facility to evaluate for low bone mass. Her dual-energy x-ray absorptiometry scan was within normal limits, with a T-score of 1.2 in the L1 to L3 region, a T-score of 1.3 in the left femoral neck, a T-score of 1.4 in the total left femur, a T-score of 0.9 in the right femoral neck, and a T-score of 1.1 in the total right femur.

**Table:** Patient Laboratory values

Date	Alkaline phosphatase, total (reference range, 31-125 U/L)	Alkaline phosphatase, bone-specific (reference range, 5.3-19.5 μg/L)	Vitamin B6 (reference range, 2.1-21.7 ng/mL, 20-125 nmol/L^a^)
February 2023	60 U/L	4.4 μg/L	43.9 ng/mL
July 2021	56 U/L	…	202.6 nmol/L^a^
March 2021	36 U/L	…	…
January 2021	50 U/L	…	301.3 nmol/L^a^
May 2020	43 U/L	…	…
December 2018	54 U/L	…	…
November 2017	34 U/L	…	…

a Different units recorded due to being performed and reported from an outside facility

She was on celecoxib, quetiapine, and amphetamine-dextroamphetamine for attention-deficit hyperactivity disorder and suspected fibromyalgia. She reported several family members to have similar dental and bone problems. Specifically, her mother was noted to have “bad teeth” that “crumbled.” Her 4 aunts had symptoms of short stature, several nontraumatic fractures, dental problems, and trigger fingers. Her maternal grandmother had lost all her teeth by the time she was in her 50s, and her maternal uncle died 2 days after birth due to prematurity and pulmonary insufficiency.

Her physical examination was unremarkable, except for a splotchy rash around the neck at initial presentation. No other findings of significance were noted.

She had an ALP level of 60 U/L (reference range, 31-125 U/L) and a vitamin B6 level of 43.9 ng/mL (reference range, 2.1-21.7 ng/mL). However, her bone-specific ALP was 4.4 μg/L (reference range, 5.3-19.5 μg/L), which prompted genetic testing. Genetic testing then confirmed a positive pathogenetic variant of the *ALPL* gene. Specifically, she had the c.542C>T (p.Ser181Leu) variant. Her 6-minute walk test demonstrated mild functional impairment with a 6-minute walk distance of 400 m, which was 60.3% of the predicted distance. Her gas exchange was adequate, as her oxygen saturation remained at or above 95% on room air during testing. Based on her history and genetic confirmation, the patient started asfotase alfa therapy to improve her symptoms and quality of life.

## Discussion

HPP is a rare disease that has different classifications and severity.^1^ The disease’s presentations range from stillbirth to adult-onset, with findings of lower-extremity fractures, premature loss of teeth, and musculoskeletal pain or incidental finding of low serum ALP.^6^ The patient presented with vague symptoms of joint pain, fatigue, and weakness when previously evaluated by other specialists to determine the source of her symptoms. Given her dental history and extensive family history, there was a strong suspicion for HPP despite her previous normal total ALP levels. Additionally, several of the patient’s family members, including the patient, had dental issues, which were consistent with HPP symptoms. After the patient was diagnosed with HPP, her mother was genetically tested and found to have the exact same genetic variant and symptoms. Specifically, premature loss of primary teeth is the most prevalent complication of HPP.^4^ Both dental and fracture history is pertinent in patients with potential HPP because ALP is a key enzyme responsible for teeth and bone formation.^7^ The patient’s uncle died from prematurity and pulmonary insufficiency, suggestive of perinatal HPP.

Although her signs and symptoms, extensive family history, and genetic confirmation were all consistent with HPP, the patient’s total ALP levels were within normal limits. This presentation is inconsistent with current literature that suggests HPP can be diagnosed based on clinical presentation and a low serum ALP. In this patient, it was not her total ALP but rather her bone-specific ALP levels that were low.

Because ALP is derived from both bone and the liver, other causes such as fractures or cholestatic liver disease may result in elevation of ALP level, thus masking the true low value.^8^ This case suggests the importance of considering bone-specific ALP in diagnosing HPP in suspect cases in addition to total ALP. It also suggests that some patients with HPP may have been overlooked or undiagnosed due to having normal ALP levels. In this patient’s case, previous specialists overlooked her HPP diagnosis and attributed her elevated vitamin B6 levels to diet and other causes rather than the disease. However, an elevated vitamin B6 level is found in patients with HPP because the diminished activity of tissue-nonspecific isoenzyme of the ALP gene causes extracellular accumulation of substrates such as pyridoxal 5’-phosphate, the circulating form of vitamin B6.^9^ Until the genetic confirmation of the pathogenic *ALPL* sequence variation was reported in the patient and subsequently the same sequence variation was also found in her mother shortly after, both patients were untreated and undiagnosed for several years.

Asfotase alfa is a TNAP enzyme replacement therapy currently approved for infantile, perinatal, and juvenile-onset HPP.^10^ Asfotase alfa has shown significant improvement in bone mineralization, leading to decreased bone pain and increased strength, agility, and motor function. The long-term effects of asfotase alfa therapy have not been determined in the literature, and their use for adults with retroactively diagnosed HPP has not been approved by the US Food and Drug Administration. However, because the clinical and laboratory findings in the patient mirror those of patients with HPP, it suggests that asfotase alfa may improve her quality of life.

## Conclusion

Current literature reports only needing low total ALP levels for diagnosis of HPP; however, this case highlights the significance of checking bone-specific ALP for the diagnosis of HPP in suspect cases with normal total ALP levels and suggests future investigation into the diagnostic approach to HPP.

## Consent

The authors attest that they are in compliance with human studies committees and animal welfare regulations of the authors’ institutions and Food and Drug Administration guidelines, including patient consent where appropriate.

## Disclosure

S.P. is a speaker for Alexion and AMGEN. The other authors have no conflicts of interest to disclose.
